# Diagnosis and surgical treatment of primary isolated aggressive lumbar myeloid sarcoma: a rare case report and review of the literatures

**DOI:** 10.1186/s12891-021-04066-2

**Published:** 2021-02-24

**Authors:** Cheng-Rui Bai, Xiang Li, Jing-Shi Wang, Jin-Jun Li, Ning Liu, Qi Fei, Dong Li, Yong Yang

**Affiliations:** 1 Department of Orthopedics, Beijing Friendship Hospital Affiliated of Capital Medical University, 95 Yong An Rd, Beijing, 100050 China; 2 Department of Hematology, Beijing Friendship Hospital Affiliated of Capital Medical University, 95 Yong An Rd, Beijing, 100050 China

**Keywords:** Myeloid sarcoma, Acute myelogenous leukemia, Lumbar spine, Decompression surgery, Case report

## Abstract

**Background:**

Myeloid sarcoma is a rare, extramedullary, solid tumor derived from immature myeloid cell precursors. It is most frequently accompanied by acute myelogenous leukemia, though infrequently found in non-acute myelogenous leukemia patients. The tumor may involve any part of the body, but the lumbar spine is seldom involved. The present case study aims to understand the diagnosis and surgical treatment of a rare primary isolated myeloid sarcoma of the lumbar spine causing aggressive spinal cord compression in a non-acute myelogenous leukemia patient.

**Case presentation:**

A 29-year-old man complained of an aggressive radiating pain to the lower extremities and moderate dysuria with a Visual Analogue Scale score that gradually increased from 3 to 8. Lumbar enhanced magnetic resonance imaging and computed tomography revealed a lumbar canal lesion at lumbar spine L2 to L4 with spinal cord compression. A whole body bone scan with fused single photon emission computed tomography/computed tomography demonstrated abnormal ^99m^Tc-methylene diphosphonate accumulation in the L3 lamina and spinous process. No evidence of infection or hematology disease was observed in laboratory tests.

Due to rapid progression of the symptoms and lack of a clear diagnosis, decompression surgery was performed immediately. During the operation, an approximately 6.0 × 2.5 × 1.2 cm monolithic, fusiform, soft mass in the epidural space and associated lesion tissues were completely resected. The radiating pain was relieved immediately and the dysuria disappeared within 1 week. Intraoperative pathological frozen section analysis revealed a hematopoietic malignant tumor and postoperative immunohistochemistry examination confirmed the diagnosis of myeloid sarcoma.

**Conclusions:**

The primary isolated aggressive lumbar myeloid sarcoma is rarely seen, the specific symptoms and related medical history are unclear. Surgery and hematological treatment are effective for understanding and recognizing this rare tumor.

## Background

Myeloid sarcoma (MS) is a rare, extramedullary, solid tumor derived from immature myeloid cell precursors [1]. MS is most frequently accompanied by acute myelogenous leukemia (AML) [2], though infrequently found in non-AML patients, and may further precede the diagnosis of hematology disease [3]. The tumor may involve any part of the body such as the skin, lymph nodes, and bone, but the lumbar spine is seldom involved [4–9]. The present case study aims to understand the diagnosis and surgical treatment of a rare case of primary isolated MS of the lumbar spine causing aggressive spinal cord compression in a non-AML patient.

## Case presentation

### Case history, physical examination, diagnostic imaging, and laboratory tests

A 29-year-old man complained of an aggressive radiating pain towards the lower extremities lasting for 1 month. He had no special medical history or fever but had moderate dysuria. Conservative treatment was ineffective. As the radiating pain was aggressive and the Visual Analogue Scale (VAS) [10] score gradually increased from 3 to 8, the patient had limited mobility in his daily life.

Physical Examination: The patient did not have anemia, subcutaneous bleeding, hepatosplenomegaly, or superficial lymph node enlargement. The spine appeared normal, with a slight limitation of lumbar motion and acute radiating pain that could be evoked by percussing the lumbar (L)2 to L4 spinous processes. The muscular tension, strength of the lower extremities, rectal tone, and perineal sensation were found to be normal. Additionally, the straight leg raising test and all pathological signs were negative.

**Fig. 1:**
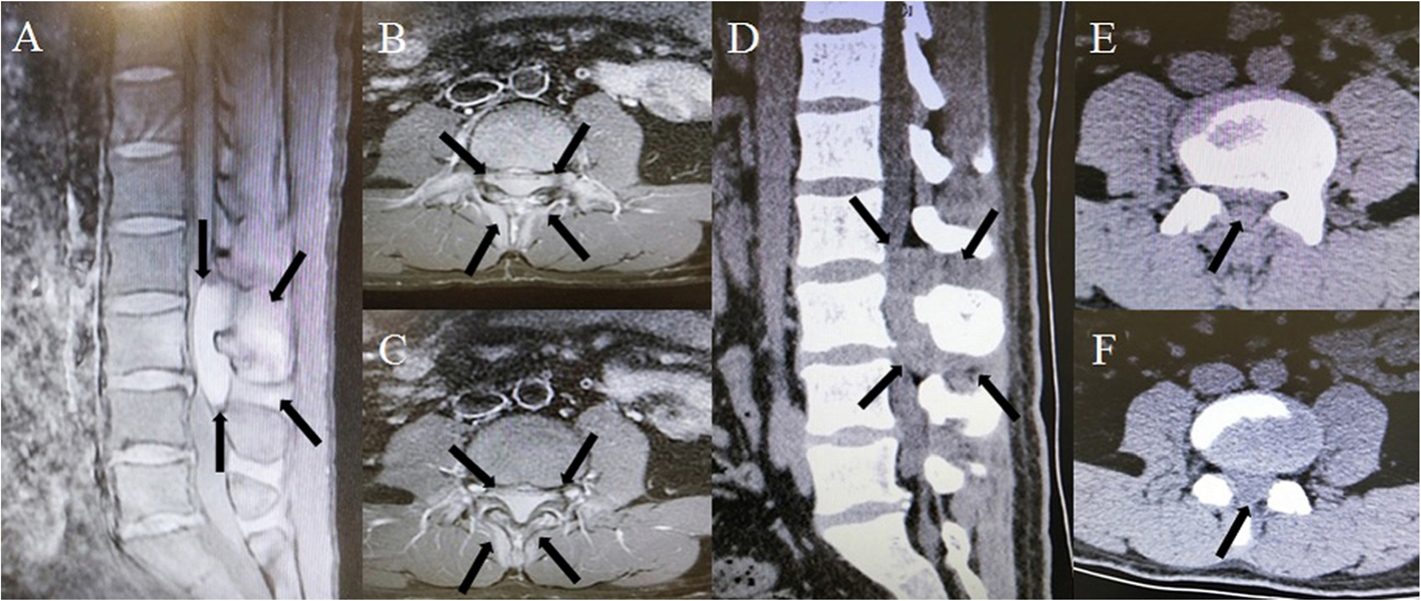
Preoperative enhanced T1-weighted magnetic resonance images (MRI) of the lumbar spine. **a** Sagittal and (**b**) (**c**) transverse views show an iso-intense, fusiform, high-signal lesion in the L2 to L4 vertebral canal, with complete compression of the local spinal cord and a mixed signal around the L3 lamina, spinous process, and soft tissue. Preoperative computed tomography (CT) of the lumbar spine. **d** Sagittal and (**e**) (**f**) transverse views show an iso-dense fusiform soft lesion at the L2 to L4 lumbar canal with local spinal cord compression and slight bone destruction.The black arrows show the scope of the lesion

**Fig. 2:**
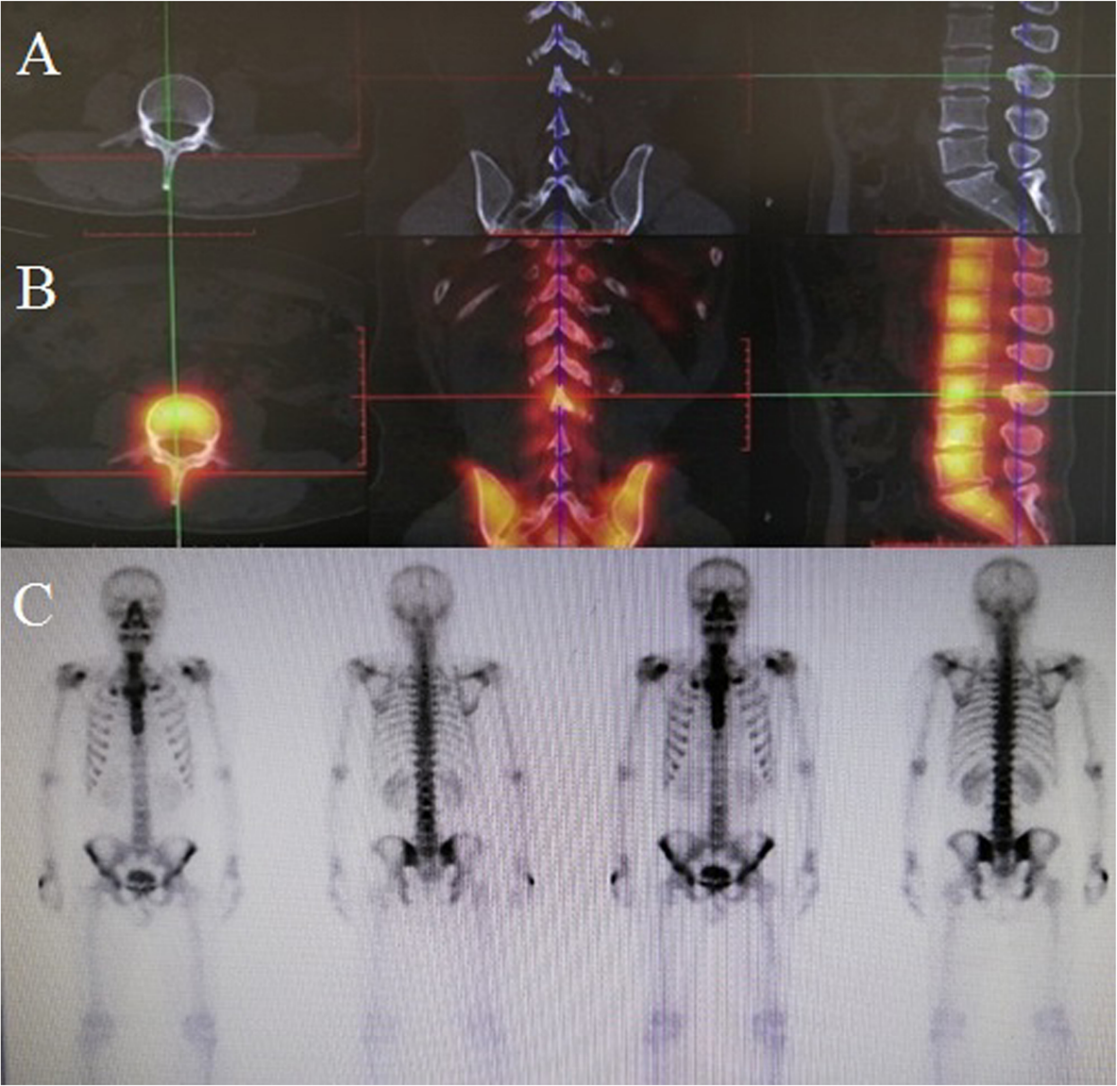
Preoperative whole body bone scan with single photon emission computed tomography (SPECT). SPECT/CT did not show bone destruction (**a**). Abnormal ^99m^Tc-methylene diphosphonate (MDP) accumulation was demonstrated in the L3 lamina and spinous process on the fused SPECT/CT (**b**) and whole body bone scan (**c**). No signs of other bone destruction and metastasis were observed in the examination

Diagnostic imaging: Lumbar enhanced magnetic resonance imaging (MRI) revealed a lumbar canal iso-intense lesion at L2 to L4 and a compressed spinal cord (Fig. 1a, b, c). A computed tomography (CT) scan of the spine showed an iso-dense, fusiform, soft lesion, and slight bone destruction at L2 to L4 (Fig. 1d, e, f). A whole body bone scan with single photon emission computed tomography (SPECT) demonstrated abnormal ^99m^Tc- methylene diphosphonate (MDP) accumulation in the L3 lamina and spinous process on fused SPECT/CT. No other signs of bone destruction or metastasis were found in the SPECT images (Fig. 2). However, as the exact character of the lesion was not clear from the images of the tumor, hematoma and abscess were not ruled out.

Laboratory tests: The components examined in the blood test were the white blood cells (WBC), 6.01 × 10^9^/L (3.50–9.50); red blood cells (RBC), 5.00 × 10^12^/L (4.30–5.80); hemoglobin (HGB), 158 g/L (130–175); platelets (PLT), 236 × 10^9^/L (125–350); C-reactive protein (CRP), 2 mg/L (0–8); erythrocyte sedimentation rate (ESR), 4 mm / 1 h (0–15); and procalcitonin (PCT), 0.27 ng/ml (0.00–0.50). Brucella tiger red experiment, T-test for tuberculosis infection, and bone marrow biopsy were found to be negative. The results from laboratory tests for infectious and hematological diseases were unclear at this point and obtaining specimens from CT or ultrasonography-assisted biopsy was unsafe and infeasible due to the highly compressed spinal cord.

### Surgery and hematological treatment

**Fig. 3:**
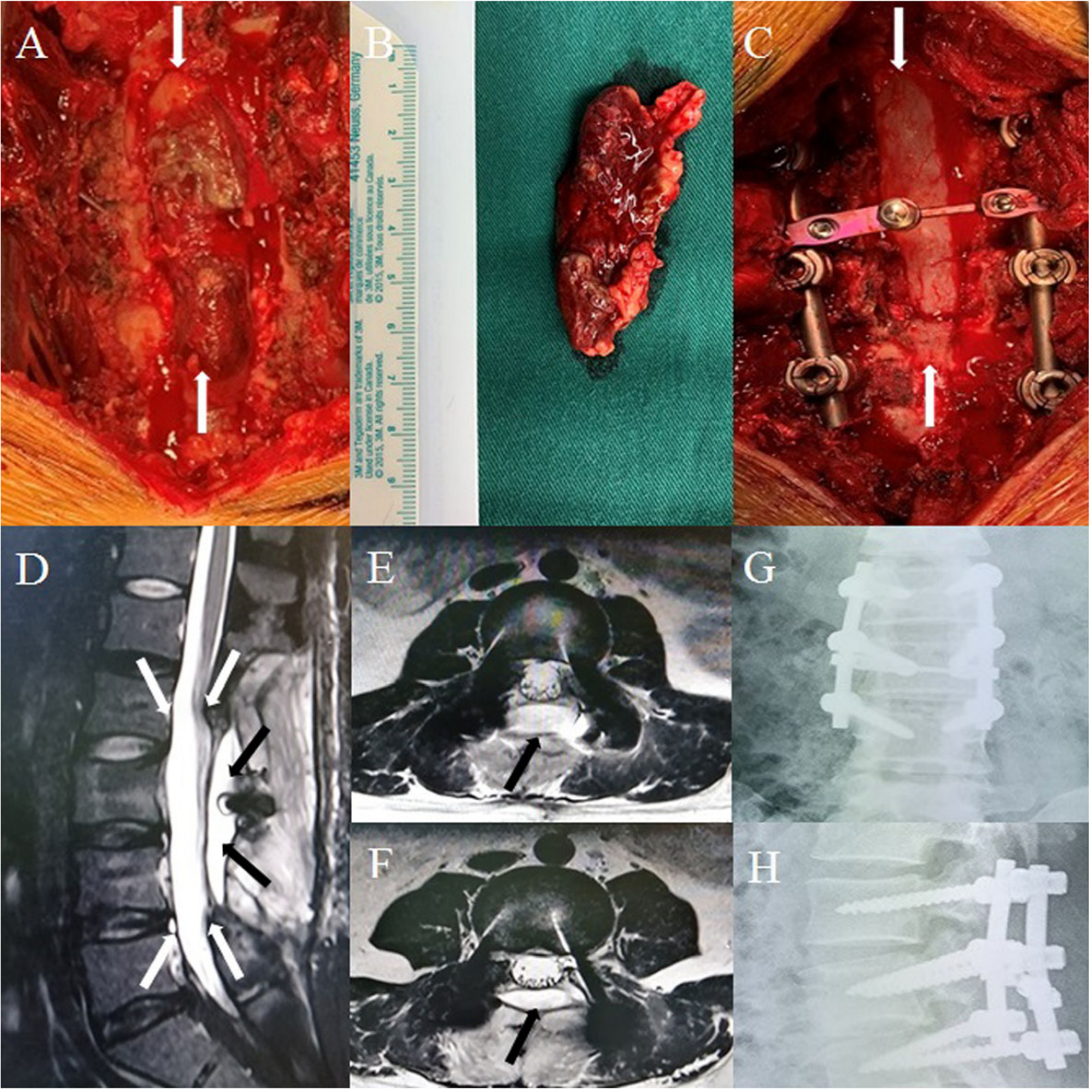
**a** The epidural soft mass in the vertebral canal during surgery. The white arrows show the scope of the mass. **b** The approximately 6.0 × 2.5 × 1.2 cm monolithic irregular fusiform soft mass was completely resected. **(c)** The restored dura sac became engorged and pulsatile after the mass resection. The white arrows show the scope of the dura sac. The postoperative 3rd month magnetic resonance images (MRI) and X-ray images. **d** T2-weighted MRI sagittal and (**e)** (**f**) transverse views show the dura sac was engorged without any compression. The white arrows show the scope of the engorged dura sac. The black arrows show the postoperative soft tissue changes. No obvious mass relapse was observed. The anteroposterior X-ray image (**g**) and lateral image (**h**) show the screw-rod fixation was normal

**Fig. 4:**
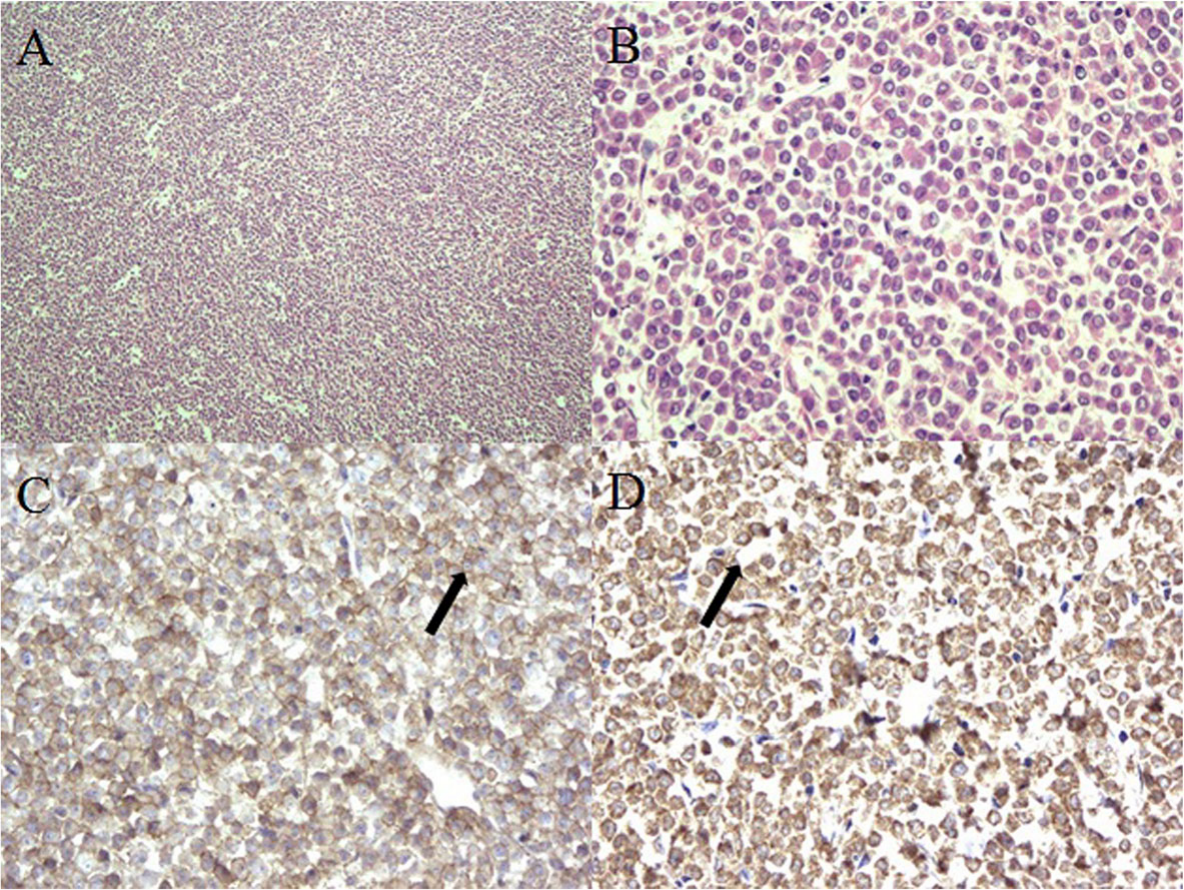
Hematoxylin and eosin-stained section. **a** Low power field view (4 × 10 magnification): diffuse, uniform, heterocytic infiltration and (**b**) high power field view (20 × 10 magnification): irregular nuclei and eosinophilic and mitotic figures are easily seen. Immunohistochemistry examination demonstrating (**c**) the positive expression of cluster of differentiation (CD)33 and (**d**) positive expression of myeloperoxidase (MPO). The diagnosis of myeloid sarcoma was confirmed. The black arrow shows the positive regions

Due to the rapid progression of the symptoms and lack of a clear diagnosis, immediate surgery was performed under general anesthesia to alleviate the symptoms and enable further determination of the diagnosis. During the posterior lumbar operation, the L3 lamina and spinous process were completely resected and the involved L2 and L4 lamina, spinous processes, and tissues were removed. In the present case, the vertebrae body was intact without a corpectomy, although the scan shows vertebrae partial involvement, the stability of the vertebrae body was not obviously destroyed. The procedure of vertebrae corpectomy indicate greater trauma, longer operation time, more bleeding, extra fixation instrument. Considering this conditions, we didn’t performed the vertebrae corpectomy. The vertebral canal was later opened and the dura sac was found intact, with no adhesions between the dura sac and mass. Subsequently, the monolithic, irregular, fusiform soft mass with approximate dimensions of 6.0 × 2.5 × 1.2 cm in the epidural space was completely resected (Fig. 3a,b). The dura sac was restored and became engorged and pulsatile immediately (Fig. 3c). During the procedure, the bone structure was partially destroyed, potentially affecting spine stability; thus, screw-rod fixation in L2-L4 was performed (Fig. 3c). A hematopoietic malignant tumor was found in the intraoperative frozen pathological section of the mass after postoperative treatment with hematoxylin and eosin (HE) (Fig. 4a, b). Further, the immunohistochemistry examination showed positive results for cluster of differentiation (CD)33, myeloperoxidase (MPO) (Fig. 4c, d), Ki67 (30% +), and CD68 (partial +). Therefore, the diagnosis of MS was confirmed based on the above positive test results.

The radiating pain was relieved shortly after the decompression operation, the VAS decreased to 2 within 1 week, and the dysuria gradually disappeared. After observing these improvements in the patient, he was transferred to the hematology department for further treatment. Although the involved vertebrae was not totally removed, the patients received chemotherapy in the sequent treatment, we hope the residual diseased vertebrae will responds to the chemotherapy [2]. Chemotherapy was performed twice in accordance with the chemotherapy protocol for AML in the hematology department. The chemotherapy regimen consisted of daunorubicin (70 mg per day) for 3 days and cytosine arabinoside (400 mg per day) for 7 days. Bone marrow transplantation was also recommended at an appropriate time after chemotherapy.

At the 3rd month of follow-up, the VAS score was 0–1, there was no mass recurrence in repeat MRI (Fig. 3d, e, f), and the screw-rod fixation was in a normal condition (Fig. 3g, h). A bone marrow biopsy showed bone marrow proliferation activity, erythroid hyperplasia, and immunotyping for CD34 (+) and CD117 (+). Early-stage myeloid cells accounted for 0.9% of the biopsy sample, with a normal proportion and phenotype. The percentage of granulocytes was 80.7%. Monocytes accounted for 3.1% and the proportion was normal, mainly comprising CD14 (+) and CD64 (+) mature monocytes. The phenotype was generally normal and the findings were consistent with no AML without bone marrow involvement. Repeat blood routine examination results were also within the normal limits. The patient was judged to be in a healthy condition. At the 10th month of follow-up, the present patient was still in a good and healthy condition with no AML without bone marrow involvement with no local lesion relapse. Indeed, the follow up time was still short, we will continue to focus on the patient, and new clinical outcomes will be reported in the future.

## Discussion and conclusions

MS, previously described as granulocytic sarcoma, myelosarcoma, or chloroma, is a rare, extramedullary solid mass composed of immature myeloid cell precursors [1, 8]. MS can appear in any age group, but shows a slight male predominance [11]. The incidence of MS in adults is particularly low, but it is most frequently accompanied by AML [2, 12]. In the present case study, the diagnosis of AML was assessed according to the World Health Organization classification for myeloid neoplasms [13]. The diagnosis of MS in the present case was made based on the positive expression of MPO, CD33, Ki67, CD68, the above findings were the surface antigens expression and immunophenotype of immature myeloid cells. MS rarely occurs in patients without bone marrow involvement i.e., AML. The disease often appears as an isolated mass at a single site, known as primary MS, which can precede the diagnosis of hematology disease in individual cases [3, 14]. The incidence of primary isolated MS is approximately 2/1,000,000 in adults, and 0.7/1,000,000 in children [11, 12]. The skin, lymph nodes, and bones are the most commonly involved parts and lumbar spine involvement is rare [4–9]. Primary isolated MS involving the lumbar spine canal in the absence of AML is extremely rare [6, 15].

In this patient, the symptoms were nonspecific with a lack of hematological disorders, associated history, physical signs, images, and laboratory findings. Obtaining a specimen through non-surgical measures is not feasible and a definite diagnosis is difficult without a sample, particularly in primary isolated MS cases [16]. MS is often misdiagnosed as non-Hodgkin lymphoma, large cell lymphoma, and other undifferentiated carcinoma and there is a 50–75% rate of misdiagnosis without immunohistochemistry findings [9, 17, 18]. In the present case, we initially speculated about a tumor diagnosis (possibly lymphoma and other spinal cord origin tumors), hematoma, and abscess, without considering the possibility of MS at all. In this crucial situation, immediate surgery was necessary to alleviate the symptoms and also to collect a specimen. The surgical procedure was important and the diagnosis was made by means of the postoperative immunohistochemical findings [16, 18].

The optimal treatment for primary isolated MS involving the lumbar spine has not yet been clearly established and there is not enough data with prospective research findings reported in the literature [7]. From the relevant case reports data showed in Table 1, we can find that most of the cases received the treatment including surgery, chemotherapy, radiotherapy, bone marrow transplantation, or any combination of these treatments, no matter there was acute myelogenous leukemia (AML) or not. Early-stage tumor resection surgery can control the local focus and lead to a long asymptomatic period and good response to other treatments, but has no effect on the survival time [19, 20]. Therefore, surgical treatment alone is not indicated except for patients whose conditions deteriorate under conservative treatment [20].

**Table 1:** The comparison between the present case and reported spine myeloid sarcoma cases in the literatures

Authors of study, Year Reference	Gender/age (years)	Leukemia or not	Involved segment	Lesion invasion site	Imaging	Bone marrow biopsy	Laboratory test findings	Treatmentregimens	Pathology and immunohisto- chemistry examination	Prognosis
Our study Case, 2020	Male/29	No leukemia	Lumbar spine	Canal Lamina Spinous processes	X-ray MRI CT SPECT	Negative	RBC5.00x10^12^/L WBC6.01x10^9^/ L HGB158g/ L PLT236x10^9^/ L CRP 2 mg/L,ESR 4 mm / 1 h, PCT 0.27 ng/ml	Mass resection Laminectomy Spinous processes resection Screw-rod fixation Chemotherapy	MPO (+) CD33 (+) Ki67 (30% +) CD68 (partial +)	Alive Without leukemia Without tumor relapse
Buckland et al., 2001 [29]	Female /35	No leukemia	Cervical spine	Paraspinal Cervical Vertebra Canal	MRI	Negative	Unremarkable finding	Laminectomy pedicles and tumor removal (NDD) Radiotherapy Chemotherapy	CD45 (+) CD45RO (-) CD79a (-)	Alive (3-month later) No evidence of AML
Landis & Aboulafia, 2003 [30]	Male/ 29	AML	Thoracic spine	Canal Vertebra Foramina	MRI	AML	WBC 2.8 x 10^9^/ L PLT 187 x 10^9^/ L Peripheral blood smear: scattered blasts	Laminectomy Partial mass resection (NDD) Chemotherapy	CD45 (+) CD43 (+) CD34 (+) CD117 (+) CD20 (-) CD3 (-)	Alive (6-month later) CR Mass regression
Shiozawa et al., 2005 [31]	Male/2	No leukemia	Lumbar and Sacral spine	Vertebra Canal Lamina	MRI	Negative	WBC1.05 x 10^4^/μL HGB12.4g/dL PLT 2.89 x 10^5^/μL	Open biopsy operation (NDD) Chemotherapy	CD13 (+) CD33 (+) CD64 (+) CD56 (+) CD68(+) CD99 (+)	Alive Mass regressed (9-month later)
Inoue et al., 2008 [32]	Female/ 26	No leukemia	Lumbar and Sacral spine	Canal Lamina Vertebra Foramina	MRI FDG‑PET	Negative	NM	Laminectomy Mass resection (NDD) Chemotherapy BMT	CD15 (+) CD45 (+) lysosome (+)	Alive (12-month later) No tumor recurrence
Takeda et al., 2009 [33]	Male/ 13	AML	Thoracic Lumbosacral spine	Canal Lamina Vertebra	MRI	AML	WBC 1.337 x 10^4^/μL HGB13.6g/dL PLT1.43 x 10^5^/μL	Laminectomy Mass resection (NDD) Chemotherapy Allogenic BMT	CD13 (+) CD33 (+) CD34 (+) CD45 (+) CD56 (+)	Alive (18-month later) CR Mass disappeared
Antic et al., 2009 [4]	Male/ 24	No leukemia	Lumbar and Sacral spine	Canal Lamina Vertebra	MRI	Negative	WBC 4.8x10^9^/L HGB126g/L PLT182x10^9^/L	Mass resection (NDD) Chemotherapy Radiotherapy Stem cell transplantation	CD34 (+) CD117 (+) HLA-DR (+)	Alive (14-month later) Free of the disease
Amritana-nd et al., 2010 [28]	Male/ 15	No leukemia	Thoracic spine	Canal Lamina Vertebra	X-ray MRI	Negative	Mild anemia (NDD)	Mass resection Screw-rod titanium cage fixation Vertebra subtotal corpectomy Radiotherapy	CD43 (+) CD99 (+) CD3 (-) CD20 (-) CD30 (-)	Death (9-month later) Tumor spread to left thigh
Serefhanoglu et al., 2010 [34]	Male/ 22	No leukemia	Cervical Thoracic Lumbar (Brain)	No figures was shown	MRI (brain only)	No leukemia	WBC6.5 x 10^3^/ mm^3^ HGB12.9mg/dL PLT2.44 x 10^5^/ mm^3^	Chemotherapy Radiotherapy	MPO (+) CD34 (+) CD2 (-) CD3(-) CD5(-) CD20 (-) CD30(-) CD56(-) Tdt (-)	Death (due to septic shock, the time was not mentioned)
Serefhanoglu et al., 2010 [34]	Female/ 43	No leukemia	Cervical spine	No figures was shown	MRI CT PET	No leukemia	Normal (NDD)	Laminectomy Mass resection (NDD) Chemotherapy Radiotherapy	MPO (+) CD3 (-) CD5 (-) CD 7 (-) CD10 (-) CD 20 (-) CD 23 (-) Tdt (-)	Death (6-month later) (ventricular fibrillation and cardiac arrest)
Xiao et al., 2013 [35]	Female/ 34	Central nervous system leukemia	Cervical spine	Canal	MRI FDG‑PET	Negative	WBC6.39x10^9^/L HGB119g/L PLT200x10^9^/L	Surgical intervention (NDD) Chemotherapy Intrathecal injections	MPO (+) TdT (partial +) Ki67(35% +) CD20(-) CD 79a(-) CD138(-) CD15(-) CD3(-) CD5 (-)	Alive (2-month later) Tumor remission in FDG-PET
Isshiki et al., 2014 [36]	Male/ 59	AML	Thoracic spine	Canal	MRI	AML	WBC 2.3 x 10^3^/μL HGB12.3g/dL PLT9.8 x 10^4^/μL	Laminectomy Mass resection (NDD) Chemotherapy	CD25 (+) CD34(+) MPO (+) CD25 (+) CD3(-) CD10(-) CD20 (-)	Alive (4-month later) CR and Tumor size decreased
Joseph et al., 2015 [2]	Male/ 20	AML(Shwachman-Diamond syndrome)	Thoracic spine	Canal Lamina	MRI	No examinati-on	NM	Mass partial resection (NDD) Radiation therapy	CD68 (weak+) CD43 (partial+) CD117(partial+)	Death (5-month later) Tumor relapse
Lama et al., 2015 [22]	Male/24	AML	Lumbar and Sacral spine	Canal	MRI	AML	NM	Decompression Mass partial resection (NDD) Chemotherapy	CD34 (+) CD43 (+) CD99 (+) Ki67(50% +)	Alive Tumor metastasis to brain (2-year later)
Yang et al., 2016 [37]	Female/ 33	AML was considere-d	Thoracic spine	Canal Lamina	MRI CT	No examinati-on	Within normal limit (NDD)	Mass partial resection (NDD)	CD10(+) CD34 (+) CD56 (+) CD68(+) CD99(+) CD117 (+) Ki67(50% +)	Death (2-day later)
Lekovic et al., 2016 [38]	Male/ 24	Leukemia (5 years history)	Cervical Thoracic Lumbar Sacral Coccyx	Canal Lamina Vertebra	MRI CT	NM	NM	Corticosteroid medication Radiation therapy Needle biopsy	MPO (+) CD43(+) CD117 (+)CD34 (+)	Alive Tumor recurrence ( 29-month later)
Massoud et al.,2016 [24]	Female/15	AMLwas considere-d	Sacral and Thoracic spine	Canal Foramina Vertebra Presacral region	X-ray MRI	No clearly shown	Blood test: neutrophil leukocytosis mild anemia, thrombocytopenia	Lesion biopsy (NDD) Chemotherapy Allogenic BMT	Confirmed the diagnosis of spinal GS (NDD)	Alive CR Mass regression (19-month later)
McCarty & Kuo, 2017 [39]	Male/14	AML	Sacral spine (brain)	Canal Sacral foramina	MRI	AML	WBC 51 x10^9^/L HGB 113 g/L PLT 130 x 10^9^/L	Chemotherapy Radiotherapy Stem cell transplantation	NM	Alive (7-month later) Stable mass size No bone marrow relapse

*MS* Myeloid sarcoma, *NM* Not mentioned, *AML* Acute myelogenous leukemia, *NDD* No detailed data, *SPECT* Single photon emission computed tomography, *BMT* Bone marrow transplantation, *WBC* White blood cell, *RBC* Red blood cell, *HGB* Hemoglobin, *PLT* Platelets, *CR* Complete remission, *FDG-PET* Fluorodeoxyglucose (FDG) - positron emission tomography (PET),*MPO* Myeloperoxidase, *CD* Cluster of differentiation, *CT* Computed tomography, *MRI* Magnetic resonance imaging, *GS* Granulocytic sarcoma, *CRP* C-reactive protein, *ESR* Erythrocyte sedimentation rate, *PCT* Procalcitonin

Isolated MS, as a systemic disease, mostly responds to systemic chemotherapy and surgery for symptomatic MS patients may be considered before starting chemotherapy [2, 21, 22]. The incidence of AML or extramedullary relapse is significantly higher in patients who are only treated with surgery [23]. As MS is rare, the optimal time and protocol for therapy has not been established for isolated MS patients and the chemotherapy regimens are similar to those for AML [2, 24]. Moreover, radiotherapy is necessary for the treatment of isolated MS when the response to chemotherapy is inadequate as well as in cases of recurrence following bone marrow transplantation or for rapid symptom relief [25]. Furthermore, positive results have been reported in some studies for the treatment of isolated MS with bone marrow transplantation and targeted therapy [11, 23, 25]. We think that therapy regimens should be selected according to the characteristic of each case.

Although non-AML MS may have a better survival rate in comparison with AML, the presence of MS is often associated with poor prognosis [2, 7, 26]. The mean survival time for patients with MS ranges between 2.5 and 22 months and is even worse for untreated patients [22]. Hence, careful investigation, rapid and accurate diagnosis, and appropriate treatment should receive the utmost priority in cases of primary isolated MS, which may have a significant impact on the survival rate and prognosis of the patient [22, 27].

To the best of our knowledge, there are no reported cases of primary isolated MS with detailed data regarding the case diagnosis, surgical strategy, and immunohistochemical features in the lumbar spine. A review of the MEDLINE database using the descriptors “spine myeloid sarcoma”, “chloroma”, “spine granulocytic sarcoma”, “vertebra canal myeloid sarcoma”, and “vertebral canal granulocytic sarcoma” retrieved relevant articles from 2000 to 2020. Upon reviewing the articles (Table 1), 17 MS cases with spine involvement were reported in 16 articles. Although the surface antigens of immature myeloid cells in different types of AML, myeloproliferative diseases, myelodysplastic syndrome and chronic myeloid leukemia are abnormal myeloid antigen expression, they are not completely consistent due to different diseases and types. The immunophenotype of myeloid sarcoma was the same as above. The common positive cell surface antigens of MS include myeloperoxidase (MPO), lysozyme, CD68-kp1, CD117, CD99, CD33, CD34, CD56, CD163, TDT, CD61, CD30, blood group glycoprotein and CD4. In addition, the positive expression of CD13, CD33, CD117 and MPO often indicates myeloid differentiation of tumor cells, and the positive expression of CD14, CD163 and CD11c indicates the differentiation of monocytes. The diagnoses of the 17 previously reported MS cases were made based on the above findings. Most of these cases had multi-focus involvement of the spine, thoracic region, and sacrum, which are the most frequently involved locations, but single lumbar spine involvement was absent. Only one case of thoracic spine MS was reported and a detailed description of the diagnosis and imaging was provided, especially the surgical treatment tactics [2, 4, 22, 24, 28–39].

The differences in history, clinical manifestation, lesion morphology, surgical methods, and prognosis were also quite significant between previous cases and the present case. Compared to previous reports, primary isolated lumbar spine involvement at a single site and more comprehensive clinical data regarding the case exhibition and surgical procedure description were presented in this case study. However, further hematologic treatment was not completely provided and the final outcome was not observed before the manuscript was submitted due to the short follow-up period in the present case. This is the limitation of our case report.

Conclusions: As primary isolated aggressive lumbar MS is rarely seen, the specific symptoms and related medical history are unclear. Hence, accurate diagnosis is challenging. However, in the present case study, immediate surgery played an important role in specimen collection and the patient’s symptoms were quickly alleviated. The prognosis of primary isolated lumbar spine MS is generally poor. However, rapid and accurate diagnosis provides the basis for further treatment and appropriate hematologic treatment should receive the utmost priority to improve the long-term outcomes. As our case study is rare and specific, this report will improve the understanding and recognition of the rare tumor in the future.

## Data Availability

Data sharing is within the manuscript.

## Abbreviations

MS: Myeloid sarcoma
NM: Not mentioned
AML: Acute myelogenous leukemia
NDD: No detailed data
SPECT: Single photon emission computed tomography
BMT: Bone marrow transplantation
WBC: White blood cell
RBC: Red blood cell
HGB: Hemoglobin
PLT: Platelets
CR: Complete remission
FDG-PET: Fluorodeoxyglucose (FDG) - positron emission tomography (PET)
MPO: Myeloperoxidase
CD: Cluster of differentiation
CT: Computed tomography
MRI: Magnetic resonance imaging
GS: Granulocytic sarcoma
VAS: Visual analogue scale
CRP: C-reactive protein
ESR: Erythrocyte sedimentation rate
PCT: Procalcitonin
HE: Hematoxylin and eosin
